# Methodological and Physiological Study during Seed Dormancy Release of *Symplocos paniculata*

**DOI:** 10.3390/plants13111459

**Published:** 2024-05-24

**Authors:** Luhong Zhang, Qiaoyu Tang, Peiwang Li, Changzhu Li, Lijuan Jiang, Jingzhen Chen, Yunzhu Chen, Qiang Liu, Yan Yang

**Affiliations:** 1 State Key Laboratory of Utilization of Woody Oil Resource, Hunan Academy of Forestry, Changsha 410004, China; 20210100019@csuft.edu.cn (L.Z.);; 2 College of Life and Environmental Sciences, Central South University of Forestry and Technology, Changsha 410004, China; 3 Key Laboratory of National Forestry and Grassland Administration on Utilization Science for Southern Woody Oilseed, Changsha 410128, China

**Keywords:** *Symplocos paniculata*, seed dormancy release, cold stratification, GA_3_, amylase, promoting germination

## Abstract

*Symplocos paniculata* are reported to exhibit seed dormancy, which impedes its cultivation and widespread adoption. In this study, a comprehensive method was established to overcome seed dormancy by subjecting seeds to scarification in 98% H_2_SO_4_ for 10 min, followed by 1000 mg·L^−1^ GA_3_ soaking for 48 h and stratification at 4 °C for 100 days. The seed germination percentage has increased significantly, to a peak of 42.67%, though the seeds could not germinate timely by NaOH scarification. Additionally, the dynamic changes of key stored substances (proteins, soluble sugars, starches, and fats), associated enzyme activities (amylases, peroxidase, and catalase), and endogenous hormones (abscisic acid, gibberellic acid, and indole-3-acetic acid) in seeds were investigated. The results demonstrated a continuous degradation of starch and fat in *S. paniculata* seeds, while the levels of protein and soluble sugar exhibited fluctuations, which probably facilitated seed dormancy breaking through energy supply and transformation. The enzymatic activities underwent rapid changes, accompanied by a gradual decrease in ABA content within the seeds with increasing stratification time. Notably, GA_3_, GA_3_/ABA, and (GA_3_ + IAA)/ABA showed significant increases, indicating their positive regulatory roles in seed germination. This study clarified the dormancy mechanism and established an effective method for the release dormancy of *S. paniculata* seeds.

## 1. Introduction

The plant *Symplocos paniculata* is widely recognized for its extensive traditional usage across various regions in Asia, notably China, India, and South Asia [1,2,3,4]. The role of this species in various ecosystems is crucial, as it serves as a vital source of sustenance and habitat for both humans and wild life [5,6,7]. Significantly, the fruit of *S. paniculata* exhibits a high oil content (~35% crude fat in dried fruit), predominantly composed of unsaturated fatty acids, rendering it suitable for utilization as an edible vegetable oil in certain mountainous regions of China [8,9,10]. The seed of *S. paniculata*, however, exhibits a profound dormancy-induced state, resulting in a significantly sluggish and feeble germination capacity. Under natural conditions, the prolonged dormancy process leads to a significant loss of viability in most *S. paniculata* seeds, resulting in formidable barriers to seed propagation and severely limiting its potential for cultivation [11,12]. Therefore, it is imperative to systematically investigate dormancy release mechanisms and develop rapid methods to enhance germination of *S. paniculata* seeds.

Considerable endeavors [13,14] have been undertaken in recent decades to comprehend the pathways governing seed dormancy release, whether induced by external environmental factors or the inherent characteristics of seeds. The previous studies [7,11] have demonstrated that the seed-coat (endocarp) of *S. paniculata* seeds exhibits hardness and impermeability, while metabolic inhibitors were also detected in the endosperm. Inherent dormancy in woody species such as *Cornus florida* [15], *Pinus bungeana* [16], *Tilia henryana* [17], *Saraca asoca* [18] and *Jatropha sinensis* [19] are controlled by maternal structures and embryo physiology. This dormancy can be broken through various types of scarification and physiological changes. Studies [20,21] have also demonstrated that scarification treatment, such as immersion in H_2_SO_4_ to disrupt the hard seed shell and enhance seed coat permeability, can effectively promote germination. Chemical soaking treatment represents another efficient strategy employed for breaking dormancy [13]. Especially, exogenous hormones such as gibberellin abscisate (GA_3_) play a crucial regulatory role in the process of breaking dormancy, facilitating the transition from a dormant state to an active one [20,22]. Moreover, cold stratification (imbibition at low temperature) is one of the most commonly employed techniques to overcome dormancy and stimulate germination, significantly enhancing seed embryo development and potentially augmenting their permeability to facilitate seed coat rupture and release from dormancy, which has been proved in plants such as *Acer platanoides* [23], and *Physalis angulata* [24]. 

A series of physiological transformations and the conversion of seed reserve materials occur during the transition from dormancy to non-dormancy in seeds, exhibiting a strong correlation with the duration of dormancy and determining whether or not a seed germinates [12,25,26]. The process of seed dormancy release is intricately linked to the provision of key stored substances that are closely associated with dormancy, as well as the maintenance of a dynamic balance in energy conversion within the seed. Besides, the regulation of the activity of amylase, peroxidase, catalase and other related enzymes facilitates the breakdown of stored substances, playing crucial roles in seed dormancy release [17,27,28]. Furthermore, endogenous hormones, such as abscisic acid (ABA), GA_3_, and indoleacetic acid (IAA), play crucial roles in the regulation of seed germination and are essential for understanding the mechanisms underlying seed dormancy release [29,30,31]. However, limited research has been conducted on the field of seed dormancy in *S. paniculata* seeds, and the methods for breaking dormancy and understanding the germination mechanism remain largely unknown. 

Based on our previous findings [11], it was demonstrated that the dormancy of *S. paniculata* seeds can be largely attributed to the presence of endogenous inhibitors and a hard endocarp. Therefore, in the present study, we employed pre-treatment through scarification using a NaOH or H_2_SO_4_ solution, followed by soaking the seeds in a GA_3_ solution and subsequently subjecting them to stratification to investigate its impact on breaking seed dormancy. Furthermore, the main stored substances, associated enzyme activities and endogenous hormones were quantified to elucidate the variations during the dormant period. Our study aims to assess the impact of diverse physical and chemical treatments on seed dormancy release, determine seed germination percentage, elucidate the mechanisms underlying seed dormancy, and establish a robust foundation for the cultivation and breeding of sexual and reproductive varieties of *S. paniculata*.

## 2. Results

### 2.1. The Effect of H_2_SO_4_ or NaOH Scarification on Seed Viability and Germination

**Figure 1:**
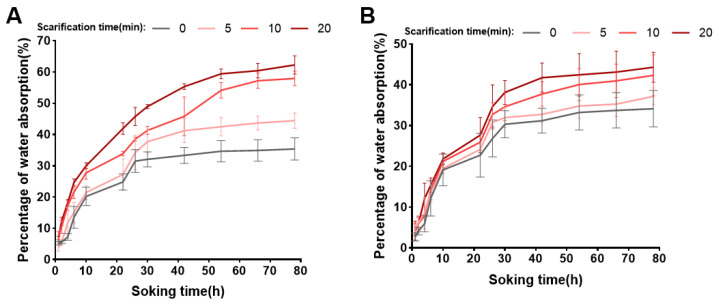
The percentage of water absorption of *S. paniculata* seeds by H_2_SO_4_ or NaOH scarification. (**A**) The percentage of water absorption varying time by H_2_SO_4_ scarification. (**B**) The percentage of water absorption varying time by NaOH scarification.

To break the seed-coat barrier, the scarification treatments of H_2_SO_4_ or NaOH were conducted on *S. paniculata* seeds. The findings unveiled that, as seeds were subjected to H_2_SO_4_ for 0, 5, and 10 min, the germination percentage exhibited a progressive increase as the scarification time increased, displaying significant differences (Table 1). Moreover, their viabilities consistently maintained high levels (>80%). When scarification time increased to 20 min, *S. paniculata* seeds exhibited a complete failure of germination and a significant decline in seed viability (viability percentage changed from 83.33% to 61.33%). Furthermore, the water absorption of seeds was assessed following H_2_SO_4_ scarification to evaluate their imbibition capacity. The percentage of water absorption for *S. paniculata* seeds in a saturated state were 44.48%, 57.92%, and 62.24% after being treated in H_2_SO_4_ for 5, 10, and 20 min, respectively (Figure 1A). Notably, the germination percentage of *S. paniculata* seeds significantly increased to 11.33% after scarification in H_2_SO_4_ for 10 min. However, when scratification time prolonged to 20 min, that resulted in seed decay and a subsequent significant reduction in their vitality.

The seeds could not germinate during the varying times of NaOH scarification in this study. Moreover, with the increase in NaOH scarification time, the viability percentage of *S. paniculata* seeds still maintained high levels (>80%), and there was no significant difference among all groups. Besides, after NaOH scarification, the water absorption of *S. paniculata* seeds were investigated, showing there were no significant differences among all treatment groups (Figure 1B).

### 2.2. The Effect of GA_3_ Soaking Combined with Stratification on Seed Viability and Germination

The regulation of seed dormancy release is typically influenced by chemical factors. In order to create optimal conditions for releasing dormancy, including subsequent H_2_SO_4_ scarification, the seeds were soaked in GA_3_ with different concentrations (water, 500, 800, and 1000 mg·L^−1^), combined with a stratification of various durations (0, 20, 40, 60, 80, 100, and 120 days). 

**Figure 2:**
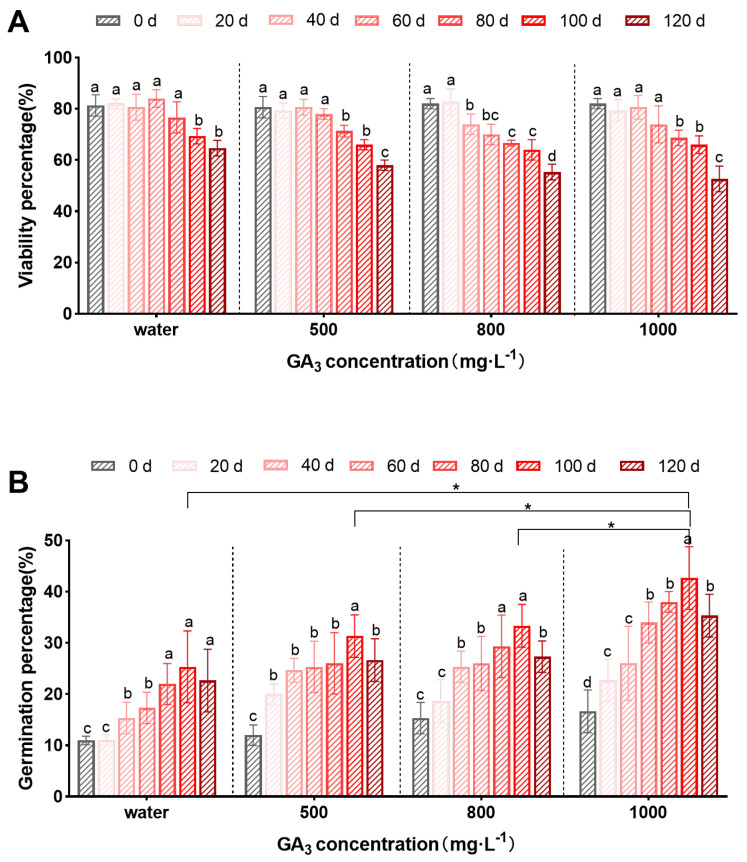
The effect of GA_3_ soaking combined with stratification on seed viability and germination. (**A**) Viability percentage. (**B***)* Germination percentage. Different lowercase letters in the same GA_3_ concentration indicate significant difference (*p* < 0.05); * indicates significant difference in GA_3_ soaking treatments.

The seed viability was influenced by stratification time and also slightly changed among various concentrations of GA_3_ soaking (Figure 2A). The results demonstrated that the viability percentage remained high in the early stages of stratification, but exhibited a declining trend in the later stages of stratification. The seed treated with 1000 mg·L^−1^ GA_3_ and subjected to 100 days of stratification was recorded at 66.00% of seed viability, which had fallen by 19.51 percent compared to non-stratified seeds (82.00%).

As depicted in Figure 2, on the other hand, for the *S. paniculata* seeds subjected to water soaking, when the time of stratification reached the 40th day, they were stimulated to germinate with a notable difference, reaching a germination percentage of 15.33%, and the highest germination percentage was 25.33% on the 100th day of stratification. Meanwhile, the seeds subjected to GA_3_ soaking without stratification showed germination percentages of 11.33%, 15.33%, and 16.67%, respectively, indicating their viability but remaining dormant. During stratification, with the increase in GA_3_ concentrations, the germination percentage of *S. paniculata* seeds increased accordingly. The germination percentage in 1000 mg·L^−1^ GA_3_ soaking was the highest on the 100th day of stratification, which was significantly different from the water, 500 and 800 mg·L^−1^ GA_3_ groups (Figure 2B). 

Moreover, with the prolongation of stratification time, the germination percentage increased accordingly in the 0 day and 100 day groups; the 100 day group performed optimally in seed germination, which was higher than other periods, and their germination percentages were raised by 2–4 times, much higher than the beginning of stratification. Overall, the seeds could effectively break dormancy under the treatment of GA_3_ and stratification, and the seed germination percentage of *S. paniculata* soaking in 1000 mg·L^−1^ GA_3_ combining with a bedding of sand for 100 days was the highest, up to 42.67%.

### 2.3. Dynamic Changes of Main Stored Substances in S. paniculata Seeds during Stratification

During seed dormancy release, the seeds are sustained by the catabolism of stored reserves like protein, soluble sugar, starch, oil and other compounds. These reserves facilitate cellular expansion within the seed and promote subsequent seedling development. Therefore, the experimental group of the highest germination percentage (H_2_SO_4_ scarification for 10 min followed by soaking in 1000 mg·L^−1^ GA_3_ and stratification), and the control group of the lowest germination percentage (H_2_SO_4_ scarification for 10 min with water soaking and stratification), were selected to investigate the dynamic differences of seed dormancy release. 

**Figure 3:**
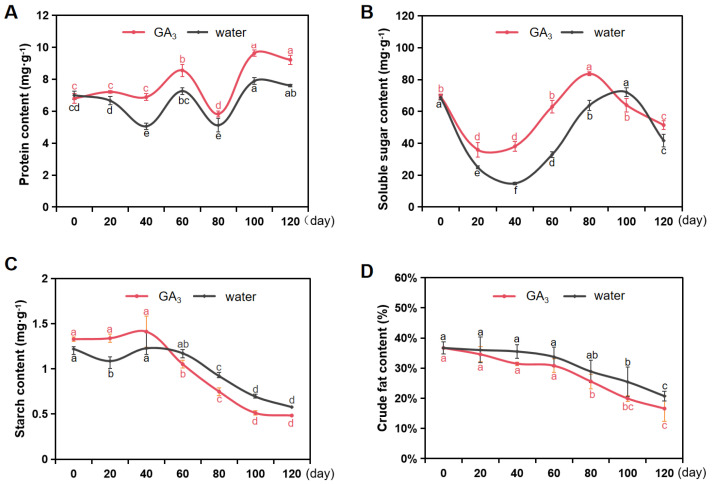
Changes of main stored substances in *S. paniculata* seeds. (**A**) Protein content. (**B**) Soluble sugar content. (**C**) Soluble starch content. (**D**) Crude fat content. Water, the treatment of H_2_SO_4_ scarification in 10 min + water soaking + stratification; GA_3_, the treatment of H_2_SO_4_ scarification in 10 min + 1000 mg·L^−1^ GA_3_ soaking + stratification. Different lowercase letters in the same treatment indicate significant difference (*p* < 0.05).

Protein is an indispensable component in the process of seed germination, and its content variation is somewhat related to the vigor of seed metabolism. The alterations in protein content between water soaking and GA_3_ soaking for *S. paniculata* seeds are presented in Figure 3A. The protein content of seeds with GA_3_ soaking exhibited a gradual increase in the early stage of stratification (0–60 days), followed by a decrease in the intermediate stages, and subsequently displayed an ascending trend, ultimately reaching its maximum value (9.63 mg·g^−1^) during the late stratification period (100–120 days). Compared to the seeds with water soaking, the protein content of the seeds soaked in GA_3_ was higher throughout the stratification process.

Soluble sugar not only serves as a source of energy and metabolites, but also plays a crucial role in establishing the osmotic gradients that regulate seed germination and development. In our study, the soluble sugar content exhibited a declining trend in the initial stage of stratification (0–40 days) in both the water soaking and GA_3_ soaking of *S. paniculata* seeds, followed by an upward rebound between 40 and 100 days of the stratification process. Notably, throughout the stratification period of 0–80 days, the seeds treated in GA_3_ soaking consistently displayed a relatively higher level of soluble sugar content compared to the seeds treated in water soaking (Figure 3B). 

The storage of starch primarily occurs in the endosperm of *S. paniculata* seeds, which is closely associated with the release of seed dormancy. Starch undergoes hydrolysis into soluble sugars and other compounds through amylase-mediated respiration, thereby providing energy and nutrients for embryonic seed development. In our study, during the initial stage of stratification (0–40 days), the starch content of the seeds treated in GA_3_ soaking remained stable and then subsequently exhibited a significant decline in the 40–100 days stage. Notably, in the middle and late stages of stratification (60–120 days), the starch content of seeds of GA_3_ soaking was consistently lower than that observed in seeds of water soaking (Figure 3C).

The accumulation of fat serves as the primary means of energy storage in oil crops, while also playing a crucial role in providing energy during seed dormancy and germination. With the prolonged stratification, both the seeds in water soaking and GA_3_ soaking exhibited a continuous decrease in the crude fat contents of the seeds. Moreover, the fat content of seeds treated with GA_3_ soaking, on the 120th day of stratification, was found to be significantly lower than that of the seeds treated with water soaking. Specifically, the crude fat content of GA_3_-soaked seeds decreased from 36.23% to 16.20%, representing a remarkable reduction by 55.29% during stratification (Figure 3D). 

### 2.4. Dynamic Changes of Related Enzymatic Activity in S. paniculata Seeds during Stratification

**Figure 4:**
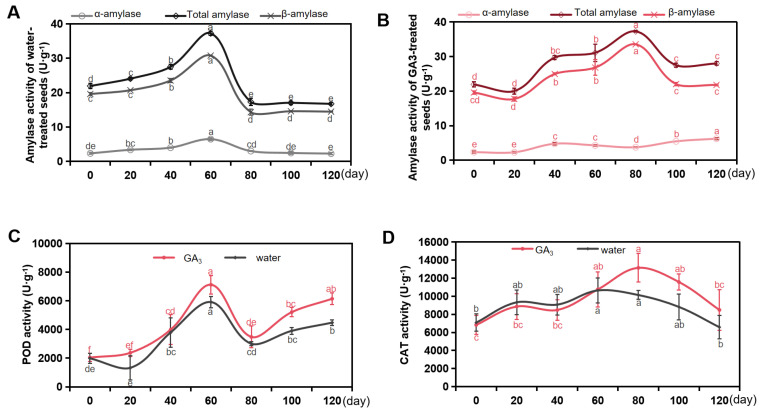
Changes of enzymatic activity of *S. paniculata* seeds during stratification. (**A**) Amylase activity of water-treated seeds. (**B**) Amylase activity of GA_3_-treated seeds. (**C**) Peroxidase (POD) activity. (**D)** Catalase (CAT) activity. Water, the treatment of H_2_SO_4_ scarification in 10 min + water soaking + stratification; GA_3_, the treatment of H_2_SO_4_ scarification in 10 min + 1000 mg·L^−1^ GA_3_ soaking + stratification). Different lowercase letters in the same treatment indicate significant difference (*p* < 0.05).

During the process of stratification, the seed embryo of *S. paniculata* seeds continuously undergoes nutrient decomposition and synthesis, which exhibits a significant correlation with amylase activity fluctuations. The dynamic change in amylase activity is characterized by complexity, as demonstrated in Figure 4A,B. Notably, β-amylase exhibited significantly higher activity compared to α-amylase during the stratification process. The α-amylase activity of GA_3_-soaked seeds exhibited a consistent increase throughout the entire stratification period, while the β-amylase activity of GA_3_-soaked seeds peaked at 30.78 U·g^−1^ on the 80th day of stratification.

POD, as a pivotal enzyme in the plant’s defense system during stress conditions, playing a crucial role in activating dormant seeds, demonstrated a significant increase in POD activity during the 0–60 days of stratification stage, followed by a sustained and elevated enzymatic activity at the later stage of stratification. After undergoing stratification for 120 days, the POD activity of *S. paniculata* seeds in GA_3_ soaking exhibited an almost threefold increase compared to the seeds in water soaking. Furthermore, at the same time of stratification, the POD activity of GA_3_-soaked seeds was found to be higher than that water-treated seeds (Figure 4C). 

CAT is frequently employed by cells to facilitate the decomposition of hydrogen peroxide. CAT typically exerts an influence on seed germination. During the process of stratification, there was an initial increase in CAT activity in the *S. paniculata* seeds (0–80 days), followed by a subsequent decrease (80–120 days). The CAT activity of GA_3_-soaked seeds was significantly higher than water-treated seeds for the seeds undergoing stratification for 100 days (Figure 4D).

### 2.5. Dynamic Changes of Endogenous Hormones Content in S. paniculata Seeds during Stratification

**Figure 5:**
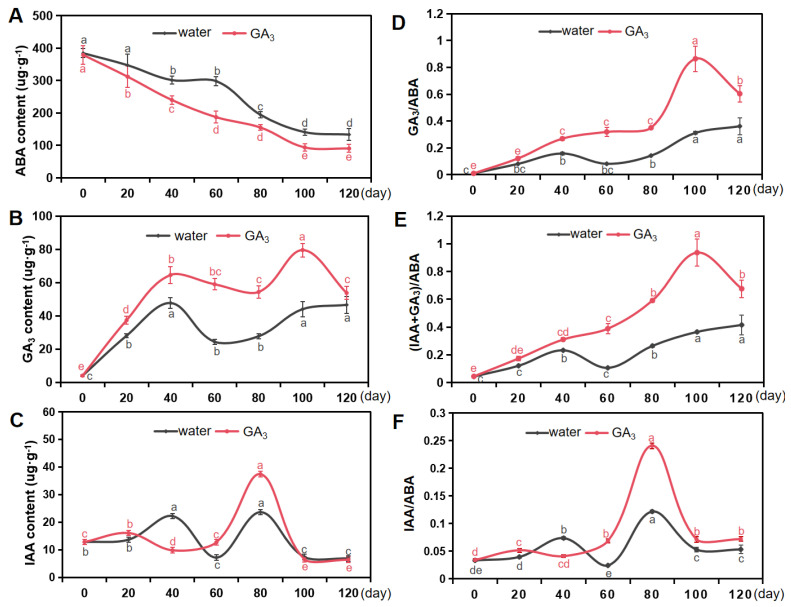
Changes of ABA, GA_3_, and IAA during stratification. (**A**) Abscisic acid (ABA) content. (**B**) Gibberellin A_3_ (GA_3_) content. (**C**) Indoleacetic acid (IAA) content. (**D**) The ratio of IAA/ABA. (**E**) The ratio of GA_3_/ABA. (**F**) The ratio of (IAA + GA_3_)/ABA. Water, the treatment of H_2_SO_4_ scarification in 10 min + water soaking + stratification; GA_3_, the treatment of H_2_SO_4_ scarification in 10 min + 1000 mg·L^−1^ GA_3_ soaking + stratification. Different lowercase letters in the same treatment indicate significant difference (*p* < 0.05).

The plant hormone ABA serves as the primary endogenous inhibitory substance, effectively suppressing seed germination and inducing seed dormancy. The ABA content of *S. paniculata* seeds, both treated in water soaking and GA_3_ soaking, exhibited a significant decline from 0 to 120 days of stratification, with a fourfold decrease compared to the beginning of statification. The minimum values of the ABA content in seeds of water soaking and GA_3_ soaking were recorded as 133.48 μg·g^−1^ and 91.16 μg·g^−1^, respectively (Figure 5A). 

During the process of stratification, the content of GA_3_ in seeds of water soaking and GA_3_ soaking showed an increasing trend. Moreover, throughout the entire stratification process, the concentration of GA_3_ consistently exceeded that of the control group on corresponding days of stratification. The GA_3_ content in GA_3_-soaked seeds especially reached a peak value of 79.01 μg·g^−1^, particularly during the stratification period of 100 days, which was nearly twice as high as that of water-treated seeds (Figure 5B).

IAA also plays a crucial role in the regulation of seed dormancy and non-dormancy, potentially facilitating the transition from a quiescent seed to a highly active seedling. During the stratification period, there was evident fluctuation in the IAA content of both the seeds treated in water soaking and GA_3_ soaking. Over a span of 80 days of stratification, the maximum IAA content recorded reached 24.25 μg·g^−1^ (Figure 5C).

The ABA hormone promotes the establishment and maintenance of seed dormancy, while GA_3_ triggers its release; thus, they exhibit antagonistic effects in regulating seed dormancy release. Therefore, we investigated the ratio of IAA/ABA, GA_3_/ABA, and (IAA + GA_3_)/ABA during seed stratification, as depicted in Figure 5D–F. The results revealed an oscillating increase in the IAA/ABA ratio, while the ratios of GA_3_/ABA and (IAA + GA_3_)/ABA showed a progressive rise with prolonged stratification time until reaching their maximum at 100 days. Additionally, the ABA/(IAA + GA_3_) ratio in GA_3_-soaked seeds was 0.36, whereas it was 0.94 in water-treated seeds. 

## 3. Discussion

### 3.1. Scarification Effect on Seed Dormancy Release

The impermeability of the seed coat constitutes a prevalent barrier to germination, leading to dormancy in numerous drupaceous species. This phenomenon has been substantiated by research on *Prosopis ferox* [21] and *Pyrus pyrifolia* [32], which underscore the pivotal role of the seed coat in enforcing dormancy and impeding germination. A spectrum of techniques may be implemented to surmount seed-coat-induced dormancy, encompassing the mechanical disruption of the seed coat, and chemical treatments with acids or alkalis, among other strategies.

In the case of *S. paniculata*, the seed is encased in a rigid pericarp, which acts as a physical impediment to water uptake and respiratory exchange by the embryo, thereby functioning as a barrier to its developmental progression. The current investigation examines the impact of H_2_SO_4_ and NaOH scarification. Notably, a 10 min exposure to H_2_SO_4_ was observed to effectively breach the physical dormancy of *S. paniculata* seeds, in contrast to the NaOH scarification. Furthermore, the germination percentage of seeds subjected to scarification was significantly enhanced relative to the untreated control group. However, it is prudent to highlight that increased exposure to H_2_SO_4_ may exert detrimental effects on seeds with diminished vitality.

Previous research on *Amaranthus powellii* [33] seeds has illustrated a positive correlation between increased soaking durations and germination percentage, up to a threshold of 60 s, beyond which the germination percentage declines. To disrupt the physical dormancy of *Tilia henryana* [17] seeds and stimulate germination, H_2_SO_4_ treatment was deemed a necessary precursor. Conversely, the study on *Phoebe sheareri* [34] seeds indicated that scarification treatments were not efficacious in alleviating dormancy. Within the context of the present research, the optimal scarification duration for *S. paniculata* seeds using H_2_SO_4_ was determined to be 10 min.

The findings of this study contribute to the existing body of knowledge by elucidating the optimal scarification conditions for *S. paniculata* seeds, which are critical for enhancing germination efficiency. The comparative analysis with prior research on seed dormancy and germination strategies provides a foundation for the development of evidence-based methodologies to improve seed germination outcomes. The discrepancies observed in the efficacy of H_2_SO_4_ treatment across different species may be attributed to variations in seed coat composition and thickness, which are areas deserving of further investigation. This study’s implications extend to the fields of horticulture and plant biology, offering insights into seed dormancy mechanisms and informing the development of targeted treatments to enhance germination success.

### 3.2. GA_3_ Soaking Combined with Stratification Effect on Seed Dormancy Release

Extensive research, as cited in [35,36], has established that the application of exogenous gibberellic acid (GA_3_) can significantly enhance the vitality of the seed embryo and stimulate the radicle-associated tissues, thereby facilitating the release from dormancy. In the present study, we evaluated the effects of exogenous GA_3_ treatments on the germination percentage of *S. paniculata* seeds across a range of concentrations. Our results demonstrate that seeds subjected to GA_3_ solution exhibited a marked increase in germination percentage, rising from 11.33% to 16.67%, which represents a more rapid germination percentage compared to the control group treated with water-soaked seeds. This outcome is corroborated by the findings on *Saraca asoca* seeds [18], where a GA_3_ concentration of 200 mg·L^−1^ was shown to have a pronounced effect on both seed germination and seedling growth.

In our investigation, seeds treated with a GA_3_ solution at a concentration of 1000 mg·L^−1^ emerged as the optimal group. This is in alignment with the study on *Tilia henryana* [17], which reported that exogenous GA_3_ at a concentration of 1000 mg·L^−1^ effectively promoted seed germination. It can be inferred that under the influence of GA_3_ treatment, an elevated concentration of GA_3_ is more conducive to promoting the dormancy release in seeds. 

Although chemical soaking alone is insufficient to fully alleviate the seed dormancy of *Thalictrum squarrosum* [35], our previous findings have identified inhibitors in the seed tissues surrounding the embryo as the primary contributors to physiological dormancy in *S. paniculata* seeds. The release of dormancy in this type may occur gradually or be terminated by chilling and other environmental triggers. Several studies [36,37] have demonstrated that stratification significantly influences the breaking of seed dormancy. Additionally, closely contradictory results of increases in germination percentage and decreases in viability are exhibited in our study. This may be owing to the deterioration of the physiological function of seeds during stratification, especially the qualitative change in chemical composition and the damage to cell structure.

Cold-induced dormancy is a form of physiological quiescence that persists until the seed undergoes exposure to warmer periods. Studies [25,38] have demonstrated that seeds dispersed during autumn exhibit an enhanced dormancy release following pre-treatment with stratification. The degree of dormancy varies among species and is influenced by the duration of stratification. For seeds exhibiting profound morphological and physiological dormancy, a minimum period of several months under stratification conditions is required to break dormancy [39]. In this study, the germination of *S. paniculata* seed was significantly enhanced following stratification, and the germination percentage exhibited a significant increase with prolonged stratification time. Notably, the treatment involving 100 days of stratification combined with 1000 mg·L^−^^1^ GA_3_ application resulted in a remarkable improvement compared to the water-soaked seeds, leading to a substantial elevation in *S. paniculata* seed germination to 42.67%. This observation suggests that the induction of seed dormancy release in most *S. paniculata* seeds requires an extended period of chilling, which is consistent with previous findings on seeds of *Thalictrum squarrosum* [36], *Campanula takesimana* [38], and *Thalictrum uchiyamae* [40]. The study on *Rosa damascena* [41] has also indicated that 150 days of stratification (4 ± 1 °C), following 4 weeks of warm stratification (25 °C), may be sufficient to break dormancy. However, alternative thermostatic stratification has been shown in some research to effectively release seed dormancy. Nevertheless, in comparison with the alternating thermostatic stratification, stratification is the more operationally feasible method in production, and significantly enhances the seed germination of *S. paniculata*, thereby indicating its potential for reducing the dormancy duration. This study provides crucial insights into the understanding of stratification as a method to break the dormancy of *S. paniculata* seeds.

### 3.3. Study on Main Stored Substances Changes of S. paniculata Seeds during Dormancy Release

The release of seed dormancy involves an energy-demanding equilibrium that facilitates seed germination by maintaining cellular osmotic regulation, which is closely associated with the breakdown of essential nutrients such as carbohydrates, proteins and lipids [42]. The alterations in the main stored substances of *S. paniculata* seeds elucidate the underlying mechanism by which the seed gradually transitions from a dormant state to a germinating state. 

Protein, one of the most important nutrients, is closely related to seed dormancy release as a nitrogen source supplier. The variation in protein content of *S. paniculata* seeds at the early stage of stratification was less prominent, which may be due to the fact that the state of seed dormancy still existed. The soluble protein content underwent significant changes as seed dormancy gradually released during the late stage of stratification. Subsequently, soluble proteins were continuously decomposed into small molecules such as free amino acids to provide energy, while new proteins were synthesized to support seed germination. The protein content in GA_3_-soaked seeds exhibited higher levels compared to water-soaked seeds during stratification, potentially contributing to the disruption of seed dormancy. Soluble sugars are undeniably crucial for seed imbibition and seedling development, as they can be readily absorbed for respiratory consumption during seed germination. This result demonstrated that the content of soluble sugar was actively changed in the transformation from dormant to non-dormant of *S. paniculata* seeds. The soluble sugar content firstly decreased and then increased and reached the maximum at the 80th day of stratification, which may be related to the decreasing of starch that further decomposes into soluble sugars under the catalysis of amylases. Therefore, this explains the observed increase in soluble sugar content during the later stages of stratification. The content of protein and soluble sugar aligns with the findings reported in previous studies on the seed stratification of *Phoebe sheareri* [34], *Cercis chinensis* [43], *Campanula takesimana* [38], *Michelia chapensis* [28] and *Tilia henryana* [17].

As a crucial energy reservoir in seed dormancy, starch can be hydrolyzed by amylases into smaller ingredients, such as sugars, for supplying energy during seed dormancy release. The results of this study were analyzed, revealing a gradual decomposition of starch with an increased stratification time. The starch content in particular exhibited a significant decline during the mid and late stages of stratification, while the percentage of starch decomposition was found to be faster under GA_3_ treatments compared to water-soaked seeds. Additionally, it is worth noting that *S. paniculata* seeds possess a rich crude fat content primarily stored in the endosperm. During stratification, the fat content of seeds in GA_3_ decreased from 36.23% to 16.20%, representing a significant reduction of 55.29%. This phenomenon suggests that the fat is gradually transformed into some organic small molecular substances under the stratification, thereby serving as an energy source and functional foundation for seed dormancy release. Similar studies have previously been carried out on *Cornus florida* [15] and *Jatropha curcars* [44]. 

### 3.4. Study on Related Enzymatic Activity Changes of S. paniculata Seeds during Dormancy Release

During the process of stratification, the macromolecular storage substances in seeds undergo continuous enzymatic degradation, leading to their conversion into soluble compounds that can be utilized by embryo metabolism. This provides a source of nutrients and energy for seed germination. 

The α-amylase enzyme is primarily utilized for the hydrolysis of starch and plays a crucial role in starch metabolism. Conversely, β-amylase serves as an essential catalyst for the breakdown of sugars into glucose, fructose, and other components. The amylases exhibit a distinct alteration pattern during different stages of stratification in this study, and they are continuously activated to promote metabolic activity during seed dormancy release. The activity of β-amylase was much higher than that of α-amylase during stratification. These results indicated that the process of stratification was conducive to a metabolic reaction, seemingly assisting in the starch production of soluble metabolites converted by amylases. Moreover, these substances can effectively facilitate the softening of the seed-coat in *S. paniculata*, thereby enhancing the gas–water exchange within the seeds and promoting an active respiratory pathway, ultimately expediting the transition from seed dormancy to dormancy release. The amylase facilitated the decomposition of stored substances and regulated seed dormancy release, as evidenced by studies on *Solanum tuberosum* [45], *Tilia henryana* [17] and *Paeonia ostii* [26]. 

Several early studies have reported that the seed germination was significantly regulated by antioxidant enzymes like POD and CAT [28,42], among which POD can remove excess free radicals, thereby improving the plant’s stress resistance, but can also decompose IAA by oxidation, thus affecting seed dormancy release. The enzymatic activities of POD and CAT were modulated under different environmental conditions, ensuring a balanced enzymatic environment for seed vitality. Our findings demonstrate an increasing trend in the activities of POD and CAT during stratification, with a significant enhancement observed after 120 days. Specifically, the POD activity in the experimental group was almost tripled compared to its initial level, and it also exhibited higher levels than that of the control group within the same period of stratification. The CAT activity in GA_3_-soaked seeds was observed to increase during the process of stratification (0–80 days). This enhanced activity of POD and CAT indicated a frequent occurrence of hypermetabolism during seed dormancy release, which facilitated the alleviation of seed dormancy and subsequent germination. The coordination between these two enzymes ensures the prevention of cellular damage caused by superoxidizing free radicals through the removal of reactive oxygen species, thereby facilitating seed dormancy release [46]. Besides, it enhances the activity of antioxidant enzymes and reduces the degree of membrane lipid peroxidation, thus promoting the early germination of *S. paniculata* seeds. 

### 3.5. Study on Hormones Content Changes of S. paniculata Seeds during Dormancy Release

ABA, GA_3_ and IAA have emerged as the most extensively investigated plant hormones, being prominently associated with various aspects of seed dormancy [47]. In particular, it is widely acknowledged that dormancy regulation in seeds involves the antagonistic effects of ABA and GA_3_. Numerous studies [47,48] have also demonstrated that low temperature stratification can modulate the balance between GA_3_ and ABA to alleviate seed dormancy. Some interest has been garnered in elucidating the regulatory role of ABA, GA_3_ and IAA in the dormancy release and subsequent germination of *S. paniculata* seeds. 

ABA has a dominant role in seed dormancy regulation in response to signaling genes and temperature. According to our study, the ABA content decreased during the stratification process of *S. paniculata* seeds; however, unstratified seeds remained dormant and retained a high concentration of ABA. This finding demonstrates that the decrease in ABA content promotes seed dormancy release, which is consistent with previous studies on *Tilia henryana* [17,49]. GA_3_ can also be utilized to abbreviate the dormancy period and promote seed germination, as it has been documented as a stimulant for seed germination in numerous plant species by inducing hydrolytic enzymes [50]. In this study, the GA_3_ content increased proportionally with the increase in stratification time and had a positive effect on breaking seed dormancy throughout the entire process of seed germination. The regulating of seed dormancy by IAA is mainly related to its concentration. Low-enriched IAA could promote cell division in favor of germination, while higher concentrations inhibited germination [48]. The IAA content of *S. paniculata* seeds obviously fluctuated during stratification. At the early stage of stratification, the increase in IAA content was conducive to seed germination, while a decrease in IAA content in the later stage might play a part in the release of dormancy. These results are consistent with previous studies in *Cornus florida* [15] and *Elaeis guineensis* [51], which reported similar findings. 

It was also demonstrated that seed dormancy is affected through the endogenous hormone balance of ABA, GA_3_ and IAA. While previous studies [17,27] have primarily focused on the role and physiological effects of individual hormones in seed germination, it is important to recognize that endogenous hormones interact through co-regulation rather than acting independently. Hence, we investigated the ratio of IAA/ABA, GA_3_/ABA, and (IAA + GA_3_)/ABA. It has been noted that the ratio of IAA/ABA was rising in an oscillating pattern, while the ratio of GA_3_/ABA and (IAA + GA_3_)/ABA increased with the increase in stratification time until it reached the maximum on the 100th day in our study. This effect might be mediated by a shift in ABA/GA_3_ balance in favor of ABA, achieved through the transcriptional regulation of a specific gene involved in ABA and GA_3_ metabolism and signaling.

## 4. Materials and Methods

### 4.1. Materials

The seeds were collected from the *S. paniculata* germplasm bank of Hunan Academy of Forestry, located in the city of Changsha, China (113.00° E, 28.11° N) in October of 2022. The *S. paniculata* seeds utilized in this experiment are characterized by dormancy, resulting in their failure to germinate during the initial spring (harvested in the previous autumn). 

**Figure 6:**
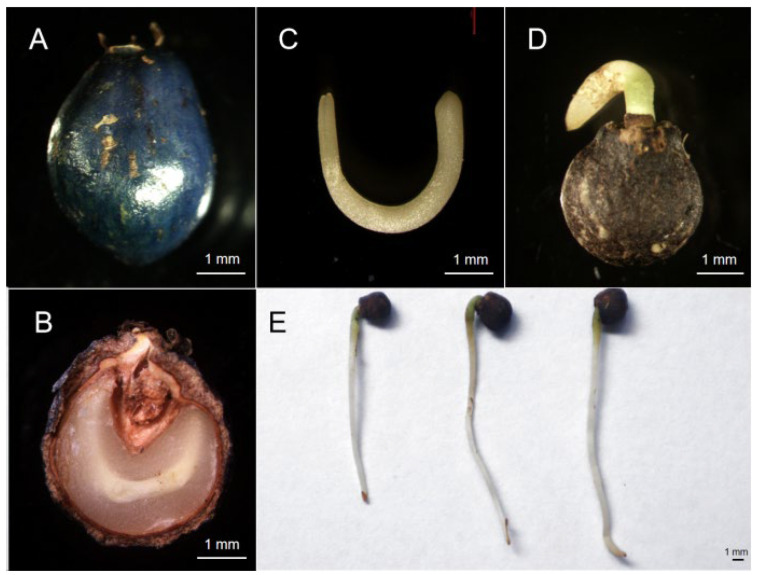
Morphological characteristics of *Symplocos paniculata* seed. (**A**) Appearance of *S. paniculate* fruit. (**B**) Cross-section of *S. paniculata* seed. (**C**) Embryo of *S. paniculata.* (**D**) Sprouting seed of *S. paniculata.* (**E**) Radicle of *S. paniculata*.

The traits of fresh, matured *S. paniculata* seeds were measured. The horizontal and vertical diameters of *S. paniculata* seeds were (4.52 ± 0.59) mm and (5.85 ± 0.61) mm, respectively. The 1000-seed mass is (47.25 ± 2.82) g. The viability percentage is ~86%. The moisture content is ~49.64%. The morphological characteristics of *S. paniculata* seeds are shown in Figure 6.

### 4.2. Methods and Treatment

#### 4.2.1. Water Imbibition Test

The investigation on the water absorption capacity of *S. paniculata* seeds was carried out in the laboratory, wherein 30 seeds per group were submerged in distilled water contained within Petri dishes (150 mm × 25 mm) lined with two layers of moistened filter paper. The seeds were weighed at intervals of 2, 4, 6, 8, 10, 22, 26, 30, 42, 54, 66, and 78 h, respectively. After a duration of 78 h, there were no further increases in seed water imibition weight.

Percentage of water absorption (%) = (mass of seeds − initial mass of seeds)/initial mass of seeds × 100%

#### 4.2.2. Seed Germination Test

The seeds were placed in Petri dishes (150 mm × 25 mm) lined with two layers of moistened filter paper, and incubated in chambers (MLR-352-PC, SANYO DENKI Co., Ltd., Tokyo, Japan) with 16 h light and 8 h dark intervals for seed germination, held at a constant temperature of 25 °C, with a humidity of 60%. Seed germination was generally counted every 3 days. Germination trials were run until no germination was detected for ten days.

The germination percentage (%) = germination seeds/total number of seeds × 100% 

#### 4.2.3. Viability Test

We used tetrazolium red (2,3,5-triphenyl-2H-tetrazolium chloride) to assess seed viability. Seeds were immersed in distilled water in Petri dishes (150 mm × 25 mm) for 30 min, then soaked thoroughly with 1% tetrazolium solution (*v*/*v*) at 25 °C for 24 h in the darkness. The viable seeds were defined only if both the embryo and endosperm were dyed in red, which was confirmed by visually observing the status of the stains showing a red color in the living tissue. 

The seed viability percentage (%) = viable seeds/total number of seeds × 100%

#### 4.2.4. H_2_SO_4_ or NaOH Scarification

The seeds were immersed in 98% H_2_SO_4_ or 30% NaOH solution for 0, 5, 10, and 20 min, respectively. Then, they were rinsed in running water for 10 min, and some seeds were put in an incubator to test the germination and viability; other seeds were placed in wet Petri dishes to conduct an imbibition test. 

#### 4.2.5. GA_3_ Soaking and Stratification Treatment

The seeds were soaked in GA_3_ with a concentration of 0 (distilled water, control group), 500, 800, and 1000 mg·L^−1^ (equal to 1.45, 2.31, 2.89 mmol·L^−1^), respectively, for 48 h. Secondly, the seeds mixed with wet sand (1:3, *v*:*v*) were put in laboratory conditions at 4 °C for stratification. Every two days in the stratification process, checked the humidity and the wet sand to maintain suitable ventilation. Every 20 days, the seeds were sampled to observe the germination and viability, and some of the seeds sampled were preserved in a −80 °C freezer for further determination. The stratification span was a total of 120 days. 

#### 4.2.6. Measurement of Seed Stored Substances Content

Protein test The protein measurements were conducted according to the previous reference [52], by using an Ultraviolet–Visible spectrophotometer under the wavelength of 595 nm.

Soluble sugar test The measurements of soluble sugar were conducted according to the previous reference [52], by using an Ultraviolet–Visible spectrophotometer under the wavelength of 630 nm.

Starch test The measurements of starch content were conducted according to the previous reference [52], by using an Ultraviolet–Visible spectrophotometer under the wavelength of 630 nm.

Fat test The crude test was conducted according to the reference [53] of the China National Standard, Determination of Fat in Food [54], applied to the Soxhlet extraction method.

#### 4.2.7. Related Enzyme Activities Determination

Amylases α-Amylases β-Amylases test The measurement of α-amylase, β-amylase, and total amylase were determined by the kit (A007-1-1, Nanjing Jiangcheng Bioengineering Institute, Nanjing, China).

Peroxidase (POD) test The measurement of POD was determined by the kit (A007-1-1, Nanjing Jiangcheng Bioengineering Institute, China).

Catalase (CAT) test The measurement of CAT was determined by the kit (BC0090, the Solibao Biological Corporation, Beijing, China).

#### 4.2.8. Determination of Hormones Content

We accurately weighed 0.2 g of seed, ground it into fine powder, added 2 mL 80% pre-chilled methanol to extract at 4 °C overnight, then centrifuged it in 5000 rpm·min^−1^ at 4 °C for 10 min, and collected the upper clear liquid. Then 80% pre-chilled methanol extraction was added to the residue, and it was centrifuged again. The collected liquid was dried using nitrogen at 4 °C, then 2 mL petroleum ether was added to decolorize the residue 3 times. The aqueous phase was extracted with ethyl acetate 3 times, and then the ethyl acetate phase was dried using nitrogen at 4 °C. The solution was then dissolved by 3.5 pH acetic acid solution, purified by C18 small columns, and methanol was used to elute. The collection of elution was reduced to dry at 4 °C, and dissolved by the flow phase, then the supernatant fluid was collected over a 0.22 μm microporous filter membrane for High Performance Liquid Chromatography (HPLC) analysis. The standards of ABA, GA_3_, and IAA were purchased from Shanghai Yuanye Bio-Technology Co., Ltd. (Shanghai, China), dissolved by the flow phase separately, and prepared into a certain concentration of standard liquid for HPLC analysis.

The determination was performed on an LC-20AT HPLC analyzer (Agilent Technologies Co., Ltd., Santa Clara, CA, USA) with an Thermo U3000 Syncronis C18 (250 mm × 4.6 mm, 5 μm); the column temperature was 35 °C; the flow rate was 1 mL·min^−1^; the injection volume was 20 uL; the mobile phase A is methanol, and the B phase is a pH 3.2 ice acetate solution, and A:B = 45:55, *v/v*; The detection wavelength of IAA and ABA was 270 nm, and the detection wavelength of GA_3_ was 206 nm. Each test was conducted with three biological replicates.

### 4.3. Statistical Analysis

The means and standard deviations of the three biological replicates were calculated using Microsoft Excel 2021. Analysis of variance (ANOVA) was performed to compare germination under different treatments using SPSS 26.0 software. Significant differences were determined using Duncan’s test at a significance level of *p* < 0.05. GraphPad Prism (Version 9.0) was utilized for data visualization. 

## 5. Conclusions

The seeds of *Symplocos paniculata* are characterized by a robust and complex dormancy mechanism, which results in an attenuated seed vigor and impeded germination. To encapsulate the findings of our study, we have demonstrated that a 10 min scarification treatment in sulfuric acid (H_2_SO_4_) significantly enhanced the permeability of the pericarp without inflicting damage upon the embryo. In contrast, seeds subjected to scarification with sodium hydroxide (NaOH) over varying durations failed to exhibit germination. The H_2_SO_4_ treatment culminated in a germination percentage of 11.33%, surpassing the performance of the NaOH treatment in terms of dormancy alleviation and enhancement of seed vitality. Moreover, the application of exogenous gibberellic acid (GA_3_) led to an increase in germination percentage from 11.33% to 16.67%.

Our study has successfully developed a comprehensive protocol for the disruption of seed dormancy in *S. paniculata*. This involves a 10 min scarification in 98% H_2_SO_4_, followed by a 48 h soak in a 1000 mg·L^−1^ GA_3_ solution, and a stratification period of 100 days at 4 °C. This method resulted in a substantial rise in seed germination percentage, reaching up to 42.67%. Throughout the dormancy release process, the seeds exhibited a progressive degradation of starch and lipid reserves, while fluctuations in the levels of protein and soluble sugars were observed. These metabolic changes are instrumental in providing the necessary energy and facilitating the transition required for dormancy breaking.

Additionally, enzymatic activities experienced rapid shifts, and the abscisic acid (ABA) content within the seeds gradually diminished over the course of stratification. In stark contrast, the ratios of GA_3_, GA_3_/ABA, and (GA_3_ + IAA)/ABA demonstrated significant augmentations, underscoring their affirmative regulatory influence on seed germination. To conclude, stratification augments seed-coat permeability, thereby promoting the translocation of nutrients within the seed and mitigating the impact of inhibitors. This is achieved through the orchestrated regulation of principal stored substances, enzymatic activities, and endogenous hormonal balances, all of which collectively contribute to the release from dormancy and the germination of *S. paniculata* seeds.

## Figures and Tables

**Table 1:** Effect of H_2_SO_4_ or NaOH scarification on germination and viability percentage of *S. paniculata* seeds.

Scarification Time (min)		0	5	10	20
H_2_SO_4_	Germination	0.00 ± 0.00 ^c^	6.67 ± 1.89 ^b^	11.33 ± 2.49 ^a^	0.00 ± 0.00 ^c^
	Viability	83.33 ± 2.49 ^a^	84.00 ± 1.63 ^a^	84.67 ± 2.49 ^a^	61.33 ± 3.40 ^b^
NaOH	Germination	0.00 ± 0.00 ^a^	0.00 ± 0.00 ^a^	0.00 ± 0.00 ^a^	0.00 ± 0.00 ^a^
	Viability	82.00 ± 3.27 ^a^	85.33 ± 2.49 ^a^	83.33 ± 0.94 ^a^	82.67 ± 3.4 ^a^

Mean values ± SEM are presented. Different lowercase letters in the same row indicate significant difference (*p* < 0.05).

## Data Availability

The datasets used and/or analyzed during the current study are available from the corresponding author on reasonable request.
